# ConSole: using modularity of Contact maps to locate Solenoid domains in protein structures

**DOI:** 10.1186/1471-2105-15-119

**Published:** 2014-04-27

**Authors:** Thomas Hrabe, Adam Godzik

**Affiliations:** 1 Program in Bioinformatics and Systems Biology, Sanford-Burnham Medical Research Institute, 92037 La Jolla, CA, USA

**Keywords:** Protein repeat detection, Solenoid structure, Contact map, Template matching, Machine learning

## Abstract

**Background:**

Periodic proteins, characterized by the presence of multiple repeats of short motifs, form an interesting and seldom-studied group. Due to often extreme divergence in sequence, detection and analysis of such motifs is performed more reliably on the structural level. Yet, few algorithms have been developed for the detection and analysis of structures of periodic proteins.

**Results:**

ConSole recognizes modularity in protein contact maps, allowing for precise identification of repeats in solenoid protein structures, an important subgroup of periodic proteins. Tests on benchmarks show that ConSole has higher recognition accuracy as compared to Raphael, the only other publicly available solenoid structure detection tool. As a next step of ConSole analysis, we show how detection of solenoid repeats in structures can be used to improve sequence recognition of these motifs and to detect subtle irregularities of repeat lengths in three solenoid protein families.

**Conclusions:**

The ConSole algorithm provides a fast and accurate tool to recognize solenoid protein structures as a whole and to identify individual solenoid repeat units from a structure. ConSole is available as a web-based, interactive server and is available for download at http://console.sanfordburnham.org.

## Background

Current estimates suggest that approximately 30% of human proteins contain multiple repeats of short motifs and could be classified as “periodic proteins” [1]. In many cases, proteins with such motifs fold into three-dimensional structures resembling solenoids (Greek *solen* (pipe) *eidos* (form)) and thus are called solenoid or solenoid-like proteins. A well-known example of solenoid proteins are Leucine Rich Repeats (LRRs) present in the innate immunity or receptors (NLR or TLR, respectively) and in thousands of other proteins with various other functions and extremely variable consensus sequences [2]. Other examples include Ankyrin repeats involved in various protein–protein interactions and Armadillo repeats that, together with other homologous classes, such as HEAT repeats, form helical solenoids and are found in proteins involved in cell adhesion [3,4].

Solenoid proteins evolved by a series of duplications of an ancestral motif, but the precise order of duplications is often unknown and may differ between and sometimes even within families. Accumulated mutations, deletions, and insertions lead to increasing divergence between individual repeats. For many proteins, this divergence can be quite extreme with almost no sequence similarity between individual copies of the ancestral motif [5]. As a result, solenoid repeats are often difficult to recognize in sequence, for instance Pfam Hidden Markov Models recognize less than half of the repeats in NLR and TLR proteins. Hence, automated detection of subtle motif variations from sequence is often impossible.

Because protein structures tend to be more conserved than sequences, similarity is retained on the structural level and recognition of the repeats is thus easier [6]. Still, repeats have significant variations of length and shape, making the precise recognition of individual solenoid units highly nontrivial. For instance, in LRR proteins the length of the individual repeats varies between 18 and 34, and not a single position, including the leucines forming the telltale pattern, is universally conserved in all repeats. The local divergence of the motifs has consequences on the global-structure level. In LRR proteins, the curvature of the entire domain varies from an ideal curvature in Ribonuclease Inhibitors (RIs) or NLRs [7] to an irregular curvature of TLRs [8], with consequences for the binding properties within the inner cavity of the protein.

Detection of repeats in proteins, both on the sequence and structure level, has gained importance as structures of more proteins with solenoid repeats have become known. Almost simultaneously, the sensitivity of sequence-based recognition has improved. Both these trends resulted in better appreciation of the relative number of proteins with repeats and the importance of the detection problem. Various detection algorithms of repeated motifs in protein sequences have been developed, with Gibbs sampling [9] and RADAR [10] as some of the first, and many others have followed [11,12]. Some of them are focused specifically on solenoid repeats in which Fourier-based analysis seems to produce the best results [13,14].

To the best of our knowledge, only four detectors of repetitive units in protein structures have been described in literature: (*i*) DAVROS [15] was probably the first method for this task, with detection based on a self-alignment matrix, (*ii*) ProStrip [16] performs repeat detection based on the C_α_ backbone angles, (*iii*) Raphael [6] is specifically devoted to the detection of solenoid repeats and is based on repeated Fourier analysis of C_α_ coordinates with appended machine-learning classification, and (*iv*) a hierarchical approach based on successive bisection of the structure into tiles for self-alignment [17]. Raphael is the best solenoid classifier to date as it significantly exceeds solenoid detection performance of sequence-based methods, while the hierarchical structural analysis from [17] is the most versatile approach to detect all possible types of structural repeats.

Here we present ConSole, a new method to determine the presence and specific positions of individual solenoid repeat units within protein structures. Template matching, a popular image-processing procedure, applied to contact maps determines whether individual residues are part of a solenoid domain or part of a non-solenoid segment. This approach is further generalized to classify whether a whole protein structure under scrutiny is solenoid or non-solenoid. ConSole is assessed on a benchmark dataset and directly compared to Raphael, the only publicly available solenoid detection algorithm. We furthermore demonstrate how accurate detection and subsequent structural alignment of solenoid units can be used to automatically retrieve the solenoid sequence motif from structure. Finally, as an example of a large-scale analysis enabled by the development of the ConSole algorithm, we analyze the length distribution of solenoid units in a large number of solenoid-like protein structures to automatically detect subtle variations of solenoid units in three solenoid protein families.

## Methods

### Pattern of solenoid units in contact maps

Contact maps (CMs) provide a simple but powerful means for protein structure comparison and alignment [18,19], prediction [20,21] and visualization of protein structural features [22]. Here we show how CMs can be used to identify solenoid proteins and to calculate lengths of individual units, even for very divergent repeats.

Below, we briefly specify the contact map definition used here and explain how solenoid unit lengths are estimated. Specifically, we define sidechains of residue *i* and *j* of all *N* residues to be in contact if the distance of any pair of their heavy atoms *a*_*i*_, *a*_*j*_ is below a specified threshold:

(1) $$c\;\left(i,\;j\right):\left|a_{i}-a_{j}\right|\;\leq \;t$$

**Figure 1:**
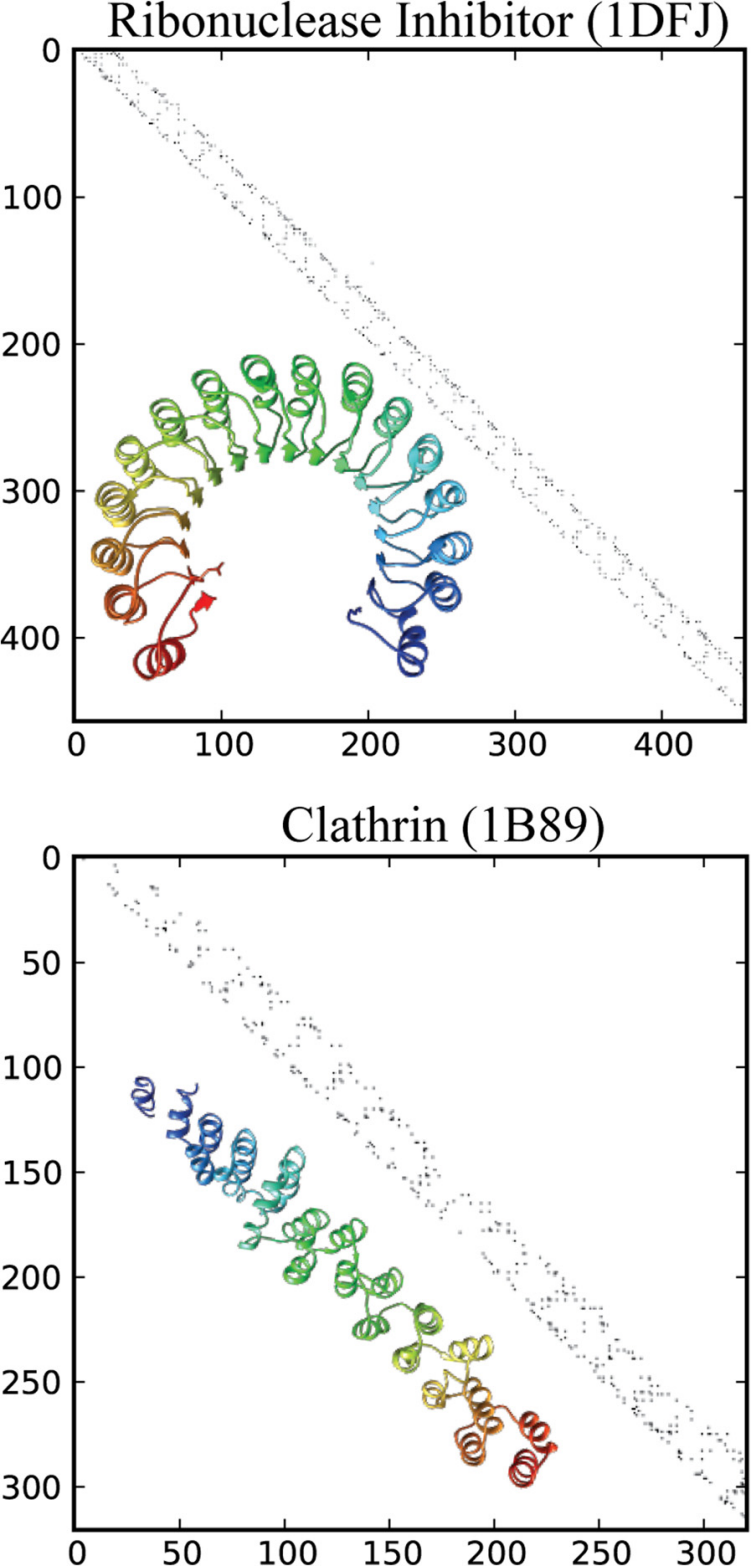
**Contact maps of solenoid protein structures.** Contact maps of two solenoid protein structures. A line *d*_*2*_, parallel to the main diagonal, is clearly visible in both CMs. *d*_*2*_ is almost fully continuous for the highly regular Ribonuclease Inhibitor (1DFJ—Chain I) and has some gaps in the Clathrin structure (1B89—Chain A), reflecting more variable interaction patterns between helices.

We use the distance threshold of *t = 4.5 Å*, following our earlier applications of contact maps [23], and assign a value of 1 (True) or 0 (False) to each of the *N* × *N* positions on the map. As expected, structural repeats in protein structures correspond to repeating patterns in the CM. The most striking feature of CMs for solenoid protein structures is an almost continuous line *d*_*2*_ of contacts running parallel to the main diagonal *d*_*1*_. The presence of a point on *d*_*2*_ indicates that a residue from one solenoid unit is in contact with a residue in the neighboring units (Figure 1). We also tested other contact definitions (C_α_, C_β_), but they did not reveal alternative significant features in the maps for solenoid detection other than *d*_*2*_.

We define *λ* as the average repeat length in a solenoid protein structure. In contact maps, the distance between *d*_*1*_ and *d*_*2*_ is related to the repeat length by a formula *λ =* | *d*_*1*_*- d*_*2*_ |. Because contacts are aligned along the main diagonal in the CM, we have to iterate along *d*_*1*_ in order to determine the most frequent contact length:

(2) $$\lambda =\;\mathrm{argma}x_{n=6}^{60}\sum_{i=0}^{N}c\;\left(i,\;i\;+\;n\right)$$

*Argmax* returns the argument with the maximum value of a function. Sampling the complete CM to obtain *λ* is not required since *λ* is expected to be in the interval *[6; 60]* of potentially contacting residues. These boundaries are based on the fact that contacts shorter than *6* residues are within the α-helical contact range and that solenoid unit lengths beyond *60* residues are virtually nonexistent [1]. Lengths of solenoid repeats are typically in the *[12; 45]* interval. Repeat lengths *λ*_*i*_ of individual solenoids unit spanning over a short segment *[i; i + λ*_*i*_*]* can also be calculated when the detection in equation 2 is confined to *[i; i + λ]*.

### Rule-based classification of solenoids vs. non-solenoids

In many solenoid proteins, regular repeats are interrupted by insertions that are not part of the solenoid. We developed a rule-based classifier analyzing only contact information to detect whether a residue is or is not a part of a solenoid unit. It mimics a human approach on detecting solenoid units in contact maps. This classifier is based on sampling each line in the contact map for each residue *i*, starting at the main diagonal *[i,i]* for each sample. The first step toward determining whether residue *i* is or is not part of the solenoid unit was to find gaps within *d*_*2*_. Gaps in *d*_*2*_ indicate insertions where the given segment does not interact with the next turn of the solenoid. We defined significant contact gaps to be at least *5* residues long, without any upper margin. A gap starting at residue *i* is defined as:¬

(3) $$\mathrm{gap}\;\left(i\right)=\mathrm{if}\;\forall j\;\in \;\left[i;\;i\;+\;5\right],\;\forall \;k\;\in \;\left[j\;+\;6;\;j\;+\;60\right]:\;\neg c\;\left(j,\;k\right)$$

In order to identify solenoid units with variable lengths, we iterate over the CM and analyze each residue *i* and the following residues within a window *I* = *[i; i + λ]*. We assign a solenoid unit starting at residue *i* by:¬

(4) $$\mathrm{solenoid}\;\left(i\right)=\;\mathrm{if}\;\forall j\;\in \;\;I:\;\neg \;\mathrm{gap}\;\left(j\right)\;\wedge \;0.5\cdot \lambda \;\leq \;\lambda_{i}\;\leq \;2\cdot \lambda$$

Then, we reassess each solenoid unit by determining the individual unit length *λ*_*i*_ with equation 2. We annotate a solenoid unit starting at *i* and ending at *i* + *λ*_*i*_ if *solenoid(i)* is true. The algorithm then continues at *i* = *i* + *λ*_*i*_ *+ 1*. If *solenoid(i)* is false, however, we continue either at *i* = *i* + *1* or at the end of a gap.

### Template matching and SVM-based classification of solenoids vs. non-solenoids

The core algorithm implemented in ConSole is based on image-processing methods to detect solenoids and non-solenoid regions in protein structures. For this, we apply a template-matching algorithm to the contact map and classify the resulting scores with a trained Support Vector Machine (SVM).

### Template matching in a contact map

Template matching (TM) is a popular image-processing technique allowing one to find specific patterns (*P*) in images (*I*) or other multidimensional data. A standard approach in TM is to use normalized cross correlation (NCC) in order to find potential areas resembling the searched pattern *P. I* and *P* must not necessarily match in size. Moreover, it is rather common that *P* is significantly smaller than *I* with accurate normalization accounting for the size difference. The NCC is typically defined as:

(5) $$\mathrm{NCC}\;{\left(I,\;S\right)}_{x,\;y}=\frac{\sum_{i,\;j}\;\left(S_{i,\;j}\;-\;\bar{S}\right)\;\left(I_{x\;+\;i,\;y\;+\;j}\;-\;{\bar{I}}_{x,\;y}\right)}{\sigma_{S}\;\sigma_{I_{x,\;y}}}$$

where $\bar{S}$ is the mean and *σ*_*S*_ the standard deviation of $S,\;{\bar{I}}_{x,\;y}$ is the mean and $\sigma_{I_{x,\;y}}$ the standard deviation of a region around *x, y* in *I* with the same size as *S*. NCC returns a matrix containing correlation coefficients in the range of *[−1; 1]*. A result of *1* indicates a perfect match, *0* indicates no similarity, and −*1* indicates inverse similarity [24].

**Figure 2:**
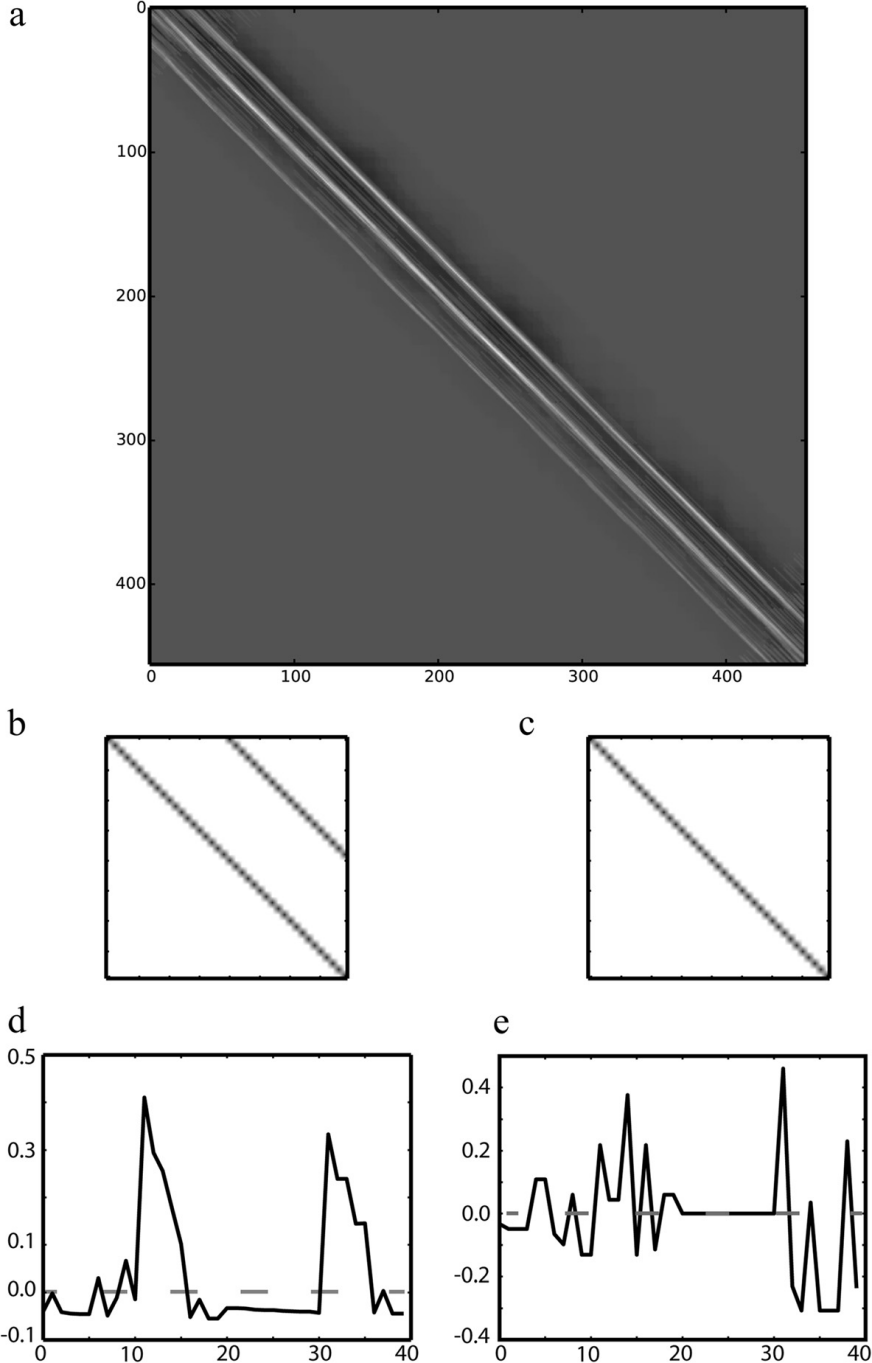
**Solenoid contact patterns. (a)** The correlation matrix *M*_*1*_ determined for template matching the contact map of PDB 1DFJ-Chain I determined with the solenoid pattern **(b)**. Bright regions indicate high correlation values [0;1], while dark regions indicate low [−1; 0] correlation values. **(b)** The solenoid pattern to generate *M*_*1*_ and **(c)** the non-solenoid pattern to generate *M*_*2*_ used for template matching. **(d)** Plot of the correlation coefficients determined for residue 75 in chain I of 1DFJ. Twenty correlation coefficients were collected around the main diagonal [(75,55); (75,95)] from *M*_*1*_ and merged with 20 coefficients from *M*_*2*_ from the same positions. **(e)** Coefficients determined for residue 81 in chain B of the globular structure from 1QGC. Correlation features determined for solenoid residues have smoother profiles compared to rather noisy features of globular proteins. Peak correlation values also differ for the solenoid/non-solenoid combinations, respectively.

Images and CMs are usually represented as a matrix of *N* × *M* pixels, and, hence, NCC can be used to localize any kind of pattern within a CM. In order to determine whether a residue is part of a solenoid or not, two patterns are correlated with the structure’s CM: (*i*) one pattern representing the typical solenoid contact pattern with two parallel diagonals, resulting in a correlation matrix *M*_*1*_ and (*ii*) one pattern with only one main diagonal for non-solenoids, resulting in a correlation matrix *M*_*2*_ (Figure 2).

Both patterns are generated dynamically at runtime. The pattern size in the *x* and *y* dimensions are set to *2λ* in order to accommodate *d*_*2*_ fully in the solenoid pattern. This way, both patterns used in the analysis are adapted to the specific solenoid length of the given structure.

### Support vector classification of correlation features

The Support Vector Machine is a machine-learning method used for supervised classification in many computational disciplines [25]. It is especially renowned for being able to classify multidimensional data while maintaining a low error rate based on its maximum margin hyper-plane determined during training.

In ConSole, we make use of the SVM to assign residues to solenoid or non-solenoid classes according to their previously determined correlation coefficients. We therefore collect correlation coefficients around the main diagonal from the NCC results as shown in Figure 2. Feature vectors are generated by concatenating 20 correlation coefficients from *M*_*1*_ with 20 correlation coefficients from *M*_*2*_ (Figure 2). All coefficients were extracted from their respective matrices at the positions *[(i,i – 10);(i,i + 10)]*. Visual inspection of the feature vectors clearly indicated significant differences between both correlation features for the same CM regions. We observed smooth correlation peaks for solenoid segments while correlation features of globular proteins had rather noisy shapes. Conclusively, the shape of the correlation peaks provides a characteristic feature for automated classification. Class labels were available from the corresponding benchmark annotation and assigned to each feature vector prior to SVM training.

### Final classification of structures

In order to compare classification results to the results in literature, we extend classification of individual residues and repeats to the level of complete structures using a simple threshold measure. If the ratio of the total number of residues classified as being in solenoid units to the number of all residues exceeds the threshold value, the whole structure is classified to be a solenoid (Equation 6):

(6) $$\mathrm{solenoid}\;\left(\mathrm{structure}\right)=\;\frac{\#\;\mathrm{solenoids}}{\#\;\mathrm{residues}}\;\geq \;t$$

Setting the *t* value to 0.5 provided the best agreement with benchmark results. A detailed assessment of different *t* values is presented in the Additional file 1.

### Detection of solenoid sequence-motifs by solenoid unit alignment

We extended the solenoid detection algorithm with an automated feature to recognize individual solenoid motifs. It is based on the local *λ*_*i*_ value where units include all residues with the indexes in *[i; i + λ*_*i*_*-1]*. We extend the usage of equation 6 to measure the quality of each solenoid unit and accept units as being solenoids only if their solenoid abundance *solenoid([i; i + λ*_*i*_*-1])* is larger than *0.75*. If *solenoid([i; i + λ*_*i*_*-1]) < 0.75* we continue with the next residue *i + 1*. This condition prevents beginnings or ends of non-solenoid regions from contributing to the motif detection.

In order to improve identification of consensus motifs, we perform structural alignment of all units using rigid alignment in the FATCAT [26] and POSA [27] pipeline. We extract the common core determined by POSA to build a sequence alignment from the respective common core overlaps [28]. Finally, we use Weblogo to visualize the consensus motif [29] for the repeat.

### Solenoid benchmark data

We used a previously published test dataset to assess the accuracy of ConSole. This dataset was originally established for testing sequence repeat detectors [13] and has since been used as a benchmark for both sequence [14] and structure based repeat detectors [6].

The benchmark comprises 105 solenoid structures for which *λ*, solenoid and non-solenoid residues, have been manually annotated. A total of 247 non-solenoid structures were also included in this dataset to provide a large variety of non-solenoid samples. The dataset contains 80,347 residues in total, out of which 19,197 were annotated as being part of solenoid repeats.

### Implementation

All the algorithms described here were implemented in Python, utilizing additional packages such as Biopython [30] for accessing PDB files, PyTom [31] for correlation functions and parallel processing on multiple CPUs, and Scikit [32] to interface with the machine-learning algorithms. The algorithm used on the server is also available for download from the server page http://console.sanfordburnham.org. Residue classification results are available in XML format containing solenoid unit boundaries for further analysis.

## Results and discussion

### Figures of merit

Based on the benchmark dataset, we were able to use annotations of (*i*) solenoid repeat lengths to evaluate solenoid length detection and (*ii*) predefined residue labels to evaluate classification results. Hence, we were able to determine true-positive (TP), true-negative (TN), false-positive (FP), and false-negative (FN) rates. Furthermore, $\mathrm{sensitivity}:\;\frac{\mathrm{TP}}{\mathrm{TP}\;+\;\mathrm{FN}},\;\mathrm{precision}:\;\frac{\mathrm{TP}}{\mathrm{TP}\;+\;\mathrm{FN}}\;\mathrm{and}\;\mathrm{accuracy}:\;\frac{\mathrm{TP}\;+\;\mathrm{TN}}{\mathrm{TP}\;+\;\mathrm{FP}\;+\;\mathrm{FN}\;+\;\mathrm{TN}}$ were determined for the solenoid and the non-solenoid class, respectively. Our final figure of merit for all algorithms was the Matthews correlation coefficient [33]:

$$\frac{\mathrm{TP}\;\times \;\mathrm{TN}\;-\;\mathrm{FP}\;\times \;\mathrm{FN}}{\sqrt{\left(\mathrm{TP}\;+\;\mathrm{FP}\right)\;\left(\mathrm{TP}\;+\;\mathrm{FN}\right)\;\left(\mathrm{TN}\;+\;\mathrm{FP}\right)\;\left(\mathrm{TN}\;+\;\mathrm{FN}\right)}}$$

### Solenoid unit length estimates

The fidelity of our solenoid detector was tested on each structure from the benchmark dataset. Each automatically detected *λ* was compared to the manually annotated value. The accuracy was determined to a mean standard deviation of *2.6* residues. The original annotators postulate that an error tolerance of up to *5* residues is acceptable for structural solenoid detectors [6], so the accuracy of our method is well within the tolerance level.

### Assessing automated solenoid classification

First, classification of residues to the solenoid or non-solenoid class was assessed for a random classifier. The underlying random distribution was adjusted to the distribution in the benchmark annotation of all residues, resulting in a distribution such that ~23% of all residues were annotated as solenoids while the remaining ~77% were non-solenoid residues.

A total of 80,347 random draws from this distribution were used to calculate the baseline performance for both of our classifiers. In the random assignments, an average of ~63% of all residues were assigned correctly while ~37% were false assignments. More precisely, 4,515 solenoids and 46,695 non-solenoids were predicted correctly. The MCC of the random residue classification was ~0.008. Extending this random assignment with equation 6 and *t = 0.33* or *t = 0.5* to the level of whole structures failed to detect any solenoid structure correctly.

Next, we assessed the classification fidelity of our rule-based classifier (Table 1). This classifier showed relatively high sensitivity and precision for solenoid residues. However, results for non-solenoid residues were low, resulting in an MCC of 0.11. Extending solenoid classification with equation 6 and *t = 0.5* to the whole structure level revealed an MCC = 0.46.

**Table 1:** Benchmark results of various solenoid classifiers

	**Rule based**		**Rule based**		**ConSole**		**ConSole**		**Raphael S > 0**	**Raphael S > 1**
	**Residue**		**Structure**		**Residue**		**Structure**		**Structure**	**Structure**
	***Sol.***	***NSol.***	***Sol.***	***NSol.***	***Sol.***	***NSol.***	***Sol.***	***NSol.***	***Sol.***	***Sol.***
**Sensitivity**	74%	40%	55%	88%	72%	88%	87%	100%	89%	93%
**Precision**	89%	19%	65%	83%	66%	90%	100%	95%	86%	98%
**Accuracy**	69%		79%		84%		96%		94%	96%
**MCC**	0.11		0.46		0.59		0.91		0.87	0.89

Evaluation of solenoid/non-solenoid classification accuracies determined for all solenoid classifiers mentioned. Sensitivity, precision, accuracy, and MCC were determined on a standard benchmark dataset of 105 solenoid (Sol.) and 243 non-solenoid (NSol.) protein structures. Results were determined either for individual residues (Residue level) or for whole structures (Structure level).

**Figure 3:**
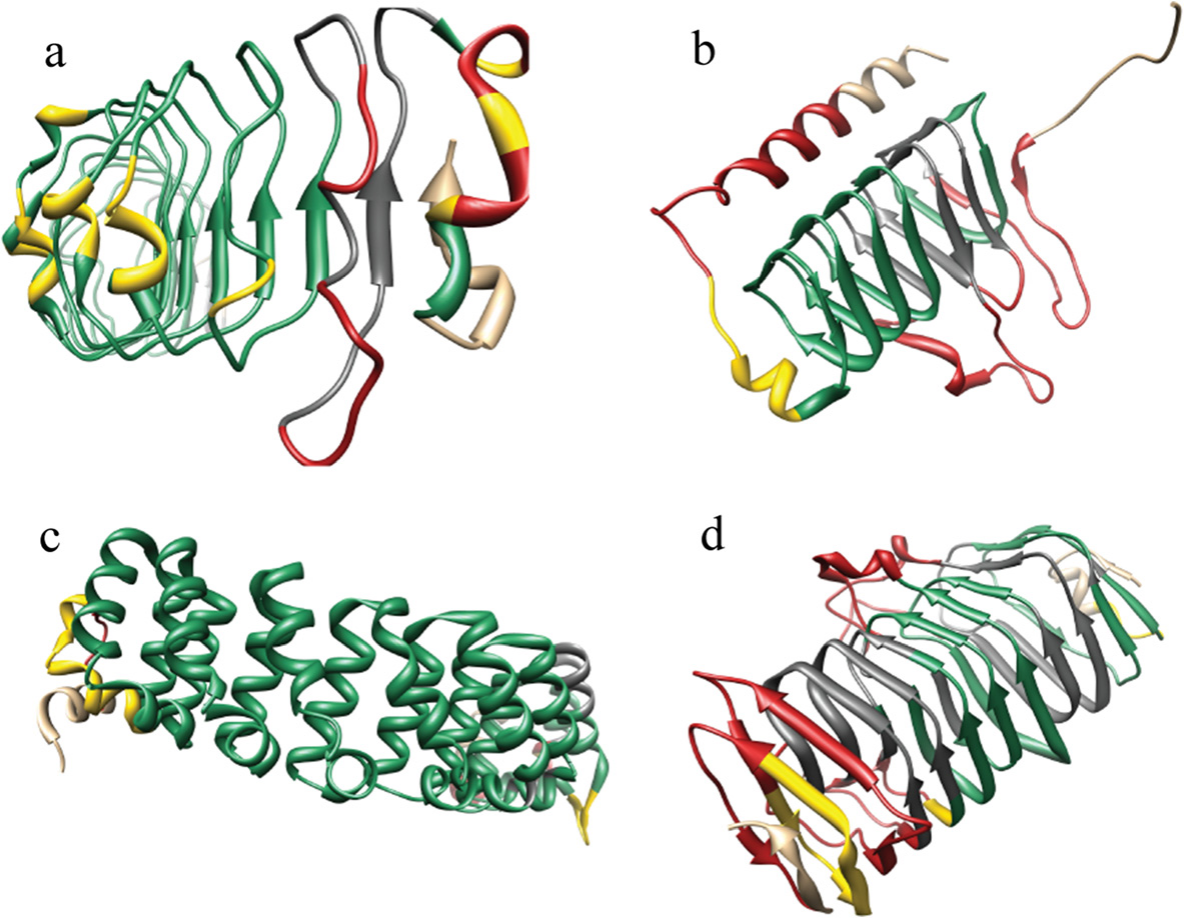
**Results of solenoid classification.** Four solenoid protein structures taken from the benchmark dataset: **(a)** 1XKU-A, **(b)** 1QRL-A, **(c)** 1M8Z-A, and **(d)** 1K5C-A. All are colored according to the ConSole-based classification results. Residues correctly assigned to the solenoid class are colored green; residues correctly assigned to the non-solenoid class are colored red. Gray or yellow indicates all residues wrongly assigned to the non-solenoid or solenoid class, respectively. Figures for all results in the benchmark are available online.

Finally, the classification fidelity of ConSole was determined at both the residue and whole-structure level (Table 1). Consistent with previous studies, a leave-one-out cross validation of the classifier was performed: all features from one structure were excluded for SVM training, and this structure was scrutinized [6]. Results of this cross validation determined the Matthews correlation coefficient for the residue classification to be 0.59. Figure 3 shows classification results of four selected structures, while all results on the benchmark data are visualized online. Details on the SVM training parameters and our trials with other classifiers (pure SVM contact classification, Decision Tree correlation classification) are provided in the Additional file 1.

In order to compare ConSole classification to other methods, we generalized classification to entire protein structures based on equation 6. Results of this generalized classification are also presented in Table 1, and the Matthews correlation coefficient was determined here to be 0.91. Based on the results published for Raphael, the MCC was determined to be 0.87 for SVM value > 0 and 0.89 for SVM value > 1.

Additional file 2: Figure S1 in the presents the ROC curve of whole-structure classification and provides an additional means for direct comparison to Raphael.

### Solenoid consensus motif from unit alignments

Detecting solenoid motifs in sequence is difficult because (*i*) the length of a solenoid repeat is typically short, increasing the signal-to-noise ratio as compared to the typical domains and full-length proteins, and (*ii*) sequence similarity may be too weak for detection of very divergent repeats. Pfam profiles for solenoid families such as LRR, Armadillo, or Ankyrin try to address these problems by defining HMMs consisting of several repeat units for divergent family members. For instance, the LRR profile for LRR1 (PF00560) has a length of 22 residues that is in accordance with the primary repeat interval [12–45]—class 3 in [34]. However, the HMM for LRR5 (PF13306) has a length of 129 residues, encompassing approximately five repeats of the actual motif. This approach is used for other solenoid families: Ankyrin HMMs: PF00023—33 residues and PF12796—89 residues (3× motif repeat), Armadillo/HEAT: PF02985—31 residues and PF13646—88 residues (3× motif repeat), and others. While improving the recognition sensitivity, this approach is inconsistent and leads to confusing results, where simultaneous high-significance matches to overlapping HMMs of different lengths are possible.

**Figure 4:**
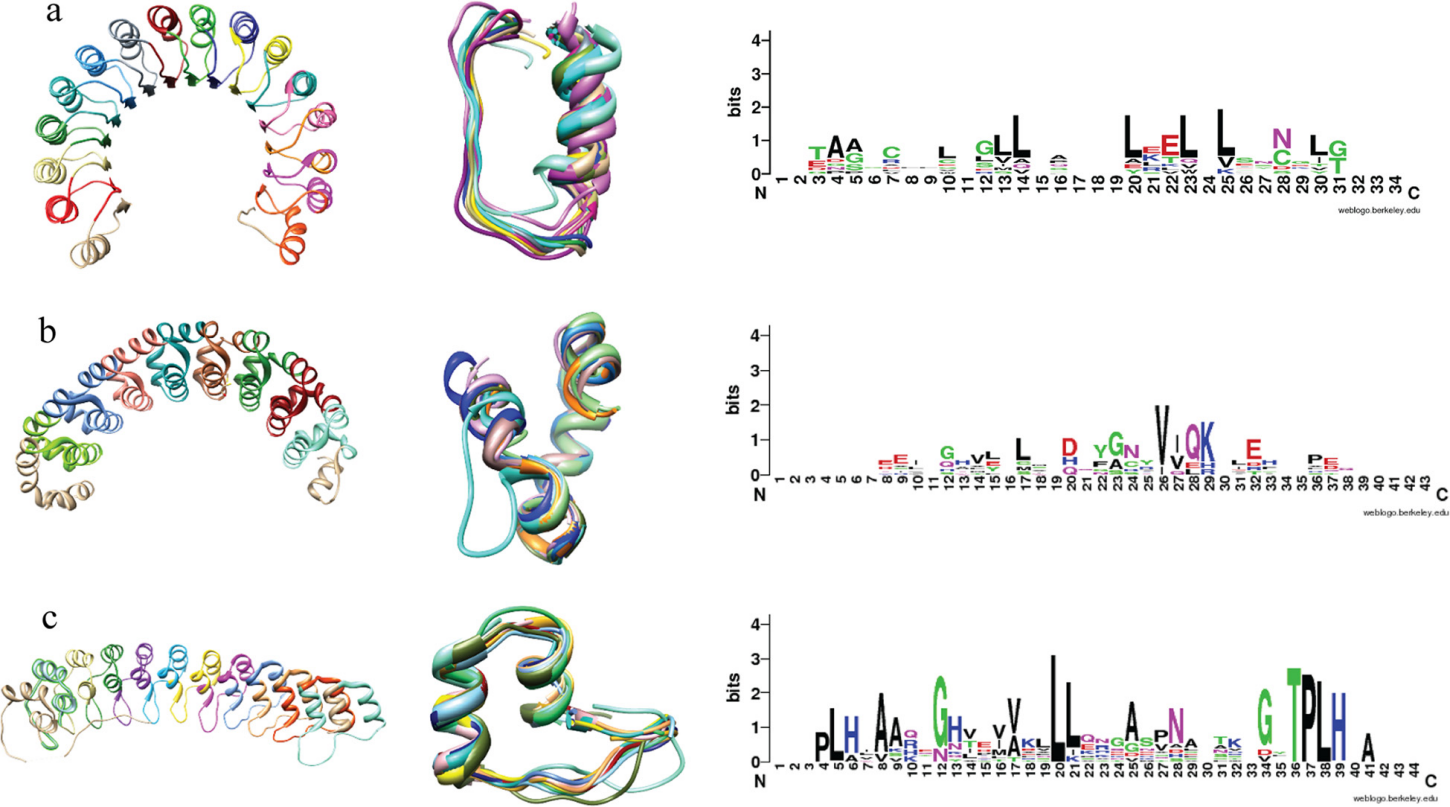
**Solenoid motif from structure alignment. (a)** Automatically detected solenoid units of a Leucine Rich Repeat domain (1DFJ-I) with arbitrary solenoid unit coloring. The middle inset shows all units superimposed after multiple-structure alignment with POSA. The consensus motif determined for the structurally aligned units is displayed on the right. The next two rows display the same results for **(b)** an Armadillo repeat (1M8Z-A) and **(c)** an Ankyrin repeat (3B95-A).

We processed structures from the LRR, Ankyrin, and Armadillo/HEAT families and determined their respective sequence motifs (Figure 4). The motifs obtained with ConSole were in excellent agreement with motifs available in literature. For instance, the sequence motif determined for the Ribonuclease Inhibitor (RI) was almost identical with the RI typical sequence [35]. The only difference to the known motif was the start position in sequence, which is an arbitrary parameter. To obtain a perfect alignment of our motif to the standard motif, the motif sequence had to be shifted by 17 residues.

### Unknown solenoid structures in the PDB

Many protein coordinate sets in the PDB are not described in a peer-reviewed manuscript and also often lack any significant annotations. To identify such proteins, we parsed all PDB headers for the keywords “JRNL REF TO BE PUBLISHED,” which resulted in a large set (16,114) of structures. In the next step, we applied ConSole to identify novel, perhaps unrecognized solenoid protein structures within this set. Indeed, 132 structures from this set were classified as solenoids.

Next, the sequence similarity of each protein against the complete collection of PDB proteins was determined. Here we ruled out homologs of proteins that have already been annotated as solenoids. The search for already known solenoid homologs was furthermore extended to the Pfam database, eliminating proteins mapping to known solenoid proteins such as Ankyrin (Pfam: 00023), Armadillo (Pfam: 00514), or Leucine Rich Repeat (Pfam: 00022).

Nineteen solenoid structures remained after these steps. Many of them were TIM barrels, identified here as solenoids because the torus-like structure also produces the second diagonal feature in contact maps. Hence, they are sometimes referred to as “closed” solenoids [1,36].

**Figure 5:**
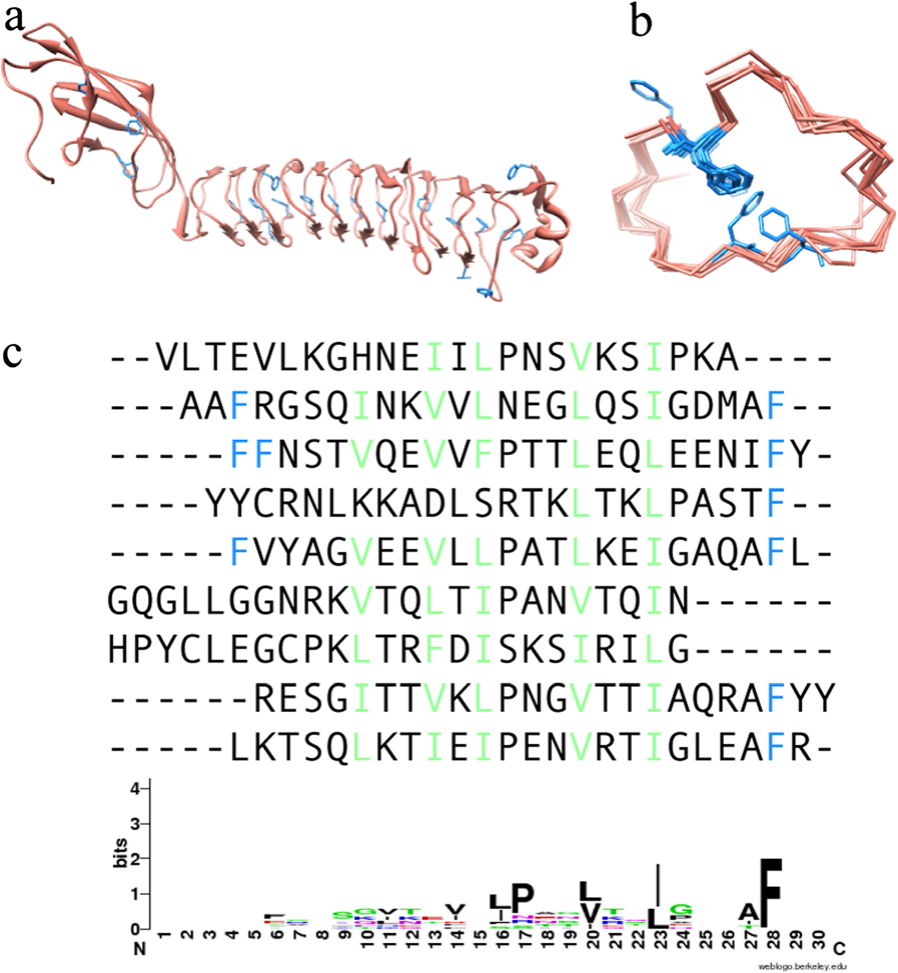
**An unusually flat LRR structure. (a)** An unusually flat LRR (4FD0-A) found within the few solenoid structures that have not yet been mentioned in publications. **(b)** Structural alignment of individual solenoid units with phenylalanine residues colored in blue. **(c)** Sequences of the respective solenoid units where all phenylalanines are highlighted in blue and all leucine-like residues are highlighted in green. Below is the sequence motif determined by ConSole.

Among the remaining true solenoid protein structures we observed were a few interesting LRR domains with an unusual flat structure (PDB: 4FD0, *Bacteriodes caccae*) or two flat domains connected by a kink (PDB: 4H09, *Eubacterium ventriosum*), both highly divergent bacterial LRR proteins. Their consensus motifs show an interesting overabundance of phenylalanine residues that could be linked to their atypical, flat structures by stacking interactions (Figure 5). The conserved region of the sequence motif determined for 4FD0 (LxxLxLxxLxxL) differs from the classical conserved motif region because there was no significant alignment of Asparagine-like residues (N) as in the standard LRR motif—(LxxLxLxxNxL). Moreover, this conserved motif matches with the TpLRR conserved sequence motif, and 4FD0 is probably one of the first structures ever to be determined for this LRR family [35].

Another unrecognized solenoid protein structure was a hypothetical protein from *B. thetaiotaomicron* (Uniprot: A7LZL0, PDB: 3N6Z). Interestingly, a domain homologous to this protein is found in one of the classes of immunoglobulin A1 proteases, where it overlaps with an N-terminal immunoglobulin A1 protease domain. This domain was not known to consist of repeats, but detailed analysis of the automatically identified repeats performed as described in the previous paragraph suggests that repeats in this domain are distantly related to GLUG repeats. GLUG is found in other classes of immunoglobulin A1 proteases, suggesting that the different classes could actually be distantly homologous.

### Analysis of solenoid unit length distributions in solenoid families

Solenoid-like protein structures, by their very nature, generally show a high degree of structural regularity. However, subtle variations at the level of individual solenoid units are possible, with accumulated mutations, deletions or insertions altering the length and shape of individual units. Such small local irregularities can add up to very significant structural differences between entire proteins and are important for functional adaptations of individual proteins.

Reliable and reproducible detection of such subtle irregularities in unit lengths for whole protein families is impossible by manual analysis. Hence, we used ConSole to automatically analyze the Leucine rich repeat, Ankyrin repeat and Armadillo repeat families for length irregularities of solenoid units. The structures were obtained from a representative set of PDB structures clustered at 90% sequence identity, a total of 140 structures (396 chains) for the LRR family, 107 structures (281 chains) for the Ankyrin and 37 structures (100 chains) for the Armadillo repeats.

**Figure 6:**
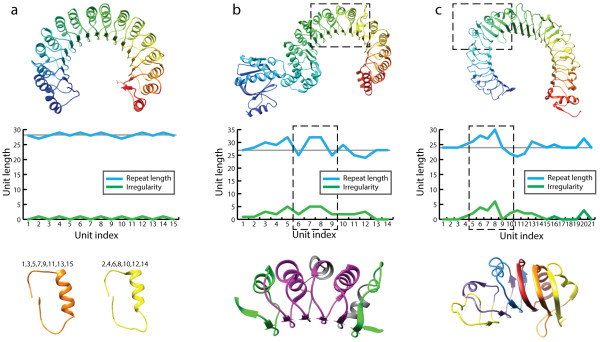
**Unit-length irregularities in Leucine Rich Repeat structures. (a)** The structure of the Ribonuclease Inhibitor (1DFJ-I) shows only minimal irregularity. The irregularity profile indicates variations in unit length *λ*_*j*_ (blue curve) and absolute deviation | *λ* – *λ*_*j*_ | from the mean unit length *λ* for each solenoid unit *j* (green curve). The two structural motifs at the bottom represent the two main structural solenoid unit motifs detected for 1DFJ. Possible positions of these structural motifs are indicated above the respective structure. **(b)** The LRR domain in CARMIL (A) resembles an unusually irregular Ribonuclease Inhibitor structure for which variations in the irregularity profile indicate variability in solenoid units. The structural segment of units 6 – 9 (dashed box) depicts irregularities in units in more detail. Each unit is colored according to the irregularity distribution in Figure 7. Gray segments were classified as insertions by ConSole and do not contribute to the irregularity calculation. **(c)** The structure of TLR4 (2Z64-A) has a distinctively more irregular region (units 5 – 10, dashed box) when compared to other segments of the structure. Irregularity in this region accrues a significant change in the curvature.

Units were assigned to irregularity classes based on the unit-length irregularity measured by |*λ*_*j*_*– λ*|, where *j* is the index of a respective solenoid unit. We now can associate length regularity profiles with the regularity of a whole structure (Figure 6). Flat profiles indicate a very regular structure, while peaks in the profile indicate positions of unusual variations in solenoid unit lengths and, as a consequence, a divergent/irregular overall structure.

**Figure 7:**
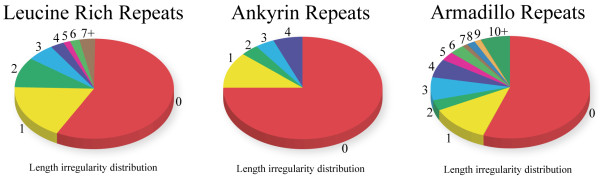
**Distribution of unit-length irregularities over several solenoid families.** Structures from the Leucine Rich Repeat, Ankyrin Repeat and Armadillo Repeat were sampled for irregularities. Displayed is the length irregularity distribution for each respective family, where the number of residues a solenoid unit length differed from *λ*.

For instance, the unit-length irregularity distribution of the LRR structures revealed that 57% of all analyzed solenoid units are highly regular (Figure 7) and, such as in the case of Ribonuclease Inhibitor (1DFJ), result in a regular, torus-like structure. However, large deviations from the mean length *λ* were observed in many structures, such as in a structure of the TLR4 extracellular domain (2Z64) where irregularities result in a horseshoe-like structure with varied curvature. This irregularity provides TLRs with the ability to adjust their shape to bind different ligands [37].

We show that Ankyrin repeats are the most regular among the three families we analyzed here, with no deviation from *λ* for approximately 75% of all solenoid units (Figure 7). On the other hand, the Armadillo repeats turns out to be the most irregular with 23% of all solenoid units being at least two residues off from the average length *λ*.

## Conclusions

In this work we present ConSole, an algorithm based on a novel combination of contact map analysis and image-processing algorithms that focuses on recognition of solenoid repeats in structures of periodic proteins.

Contact maps are naturally suited for solenoid recognition because of the presence of a characteristic line parallel to the main diagonal in the contact map. Albeit being the most intuitive approach for solenoid unit detection, direct analysis of contacts did not provide the desired accuracy of repeat recognition.

To improve the recognition, we used a standard technique of template matching in image analysis based on successive cross correlations of dynamically generated solenoid and non-solenoid patterns. The further classification of the computed correlation coefficients with a support vector machine allowed high-accuracy solenoid classification as measured by the MCC on the standard solenoid recognition benchmark.

ConSole is both more accurate and much faster than any available solenoid classifier. However, there are still a few examples of solenoid protein structures that pose challenges for the current implementation. Most notable are protein structures with non-solenoid segments running close to the solenoid domain. Such non-solenoid segments alter the contact patterns in a way that leads ConSole to classify neighboring solenoid residues as non-solenoids. An example of such a structure is the structure of gamma carbonic anhydrase (1QRL). Another factor for false classification results were false-negative classifications of complete solenoid units encapsulating long insertions (4ECO). While the insertion segment was classified correctly as a non-solenoid, residues in solenoid units in contact with the insertion were wrongly classified as non-solenoids.

One interesting application of ConSole is to analyze individual solenoid units and retrieve their consensus motifs from structural alignments. As we demonstrated, this application is robust enough to be integrated in a completely automated pipeline. We proved that separation of individual solenoid units and subsequent multiple structure alignment reliably detects solenoid specific motifs. Consensus motifs stemming from distinctive solenoid families were retrieved successfully for individual structures and indicate that current Pfam HMMs for solenoids were trained using sequences that were too long.

Finally, we extended ConSole analysis from individual structures to large groups of proteins in order to analyze the extent of structural irregularities within each family. Such local irregularities are correlated with function differences between homologs from the same family, such as a difference between Ribonuclease Inhibitor-like, regular and TLR receptors, the irregular members of the LRR family. We were also able to compare the irregularity patterns and show that Ankyrin structures generally are more regular than LRRs and Armadillo repeats.

Thus, we believe that ConSole would be useful for further sequence- or structure-based analysis of solenoid proteins as it allows the user to reliably identify consensus motifs and to detect structural irregularities, leading either to developing more accurate motif definitions or to structure analysis of individual units and detecting their variations.

### Availability of supporting data

The data sets supporting the results of this article are available in online on http://console.sanfordburnham.org in XML format and 3D visualization.

## Competing interests

The authors declare that they have no competing interests.

## Authors’ contributions

TH carried out development, implementation and assessment of the methods in ConSole. TH also prepared the figures and wrote the manuscript. AG suggested the project, critically assessed the methodological development, wrote the introduction and edited the manuscript. Both authors read and approved on the manuscript.

## Supplementary Material

Additional file 1 Parameter test for the SVM based classification approach introduced in the main article and performance assessment of alternative classifier combinations. Click here for file

Additional file 2: Figure S1 ROC curve for our solenoid detection algorithms. A receiver operating characteristic curve determined for the classification of whole structures. For the support vector machine (main article) based classifier several thresholds scanned are marked on the black curve. The decision tree based classifier (red curve) performed significantly worse. Click here for file
