# Supplementary material for: ConSole: using modularity of Contact maps to locate Solenoid domains in protein structures
Source: BMC Bioinformatics. 2014 Apr 27;15:119. doi: 10.1186/1471-2105-15-119 (PMC4021314; doi:10.1186/1471-2105-15-119)
Supplement: Additional file 1 — Parameter test for the SVM based classification approach introduced in the main article and performance assessment of alternative classifier combinations. [file 1471-2105-15-119-S1.docx]

**Additional file 1**

# Parameter tests

## SVM training parameters

In order to determine optimal SVM parameters, we processed grid search and a self-consistency test.

A random sub-set of solenoid and non-solenoid feature-vectors from the benchmark was used to train the SVM. For self-consistency, we applied the trained SVM to the same feature vectors to determine the internal errors of the machine. The very same machine was also used to classify all residues and to determine classification accuracies for different parameters. Results for the latter test are shown in the respective tables. The parameters determined were then used for running leave one out analysis of the whole dataset as described in the main article.

**Kernel type**: Multiple SVM kernel types were applied for this application:

*Radial basis functions* proved to be the best while *linear* and *polynomial kernels* showed inferior results (Table 1).

|  | **RBF** | | **Linear** | | **Polynomial** | |
| --- | --- | --- | --- | --- | --- | --- |
|  | Sol. | NSol. | Sol. | NSol. | Sol. | NSol. |
| Sensitivity | 77% | 84% | 77% | 83% | 95% | 41% |
| Precision | 61% | 92% | 59% | 92% | 33% | 96% |
| Accuracy | 83% | | 82% | | 54% | |
| MCC | 0.58 | | 0.56 | | 0.33 | |

**Supplement Table 1**: Evaluation of the SVM kernel types available in the Scikit package. RBF was detected as the best Kernel.

**C-Parameter:** The SVM-C parameter (error rate) was sampled based on a logarithmic grid search, starting with 1 – 1,000,000. The error rate finally used was determined to C=1,000. No significant improvement was detected beyond this value. Training times however significantly increased, delaying leave one out analysis *ad infinitum*. Hence, C=1,000 was used for all further steps.

The best parameters determined during this grid search were later used for the leave-one-out analysis and in the final implementation of ConSole.

|  | **C=1** | | **C=10** | | **C=100** | | **C=1,000** | | **C=10,000** | | **C=100,000** | | **C=1,000,000** | |
| --- | --- | --- | --- | --- | --- | --- | --- | --- | --- | --- | --- | --- | --- | --- |
|  | Sol. | NSol. | Sol. | NSol. | Sol. | NSol. | Sol. | NSol. | Sol. | NSol. | Sol. | NSol. | Sol. | NSol. |
| Sensitivity | 80% | 75% | 78% | 82% | 77% | 84% | 77% | 84% | 75% | 85% | 74% | 86% | 72% | 86% |
| Precision | 50% | 92% | 58% | 92% | 61% | 92% | 61% | 92% | 62% | 91% | 64% | 91% | 62% | 90% |
| Accuracy | 76% | | 81% | | 83% | | 83% | | 83% | | 83% | | 82% | |
| MCC | 0.49 | | 0.56 | | 0.58 | | 0.58 | | 0.57 | | 0.58 | | 0.55 | |

**Supplement Table 2**: Evaluation of the SVM error parameter C. No significant improvement was detected beyond C=1,000.

## Threshold estimation for classifying structures

The structures classification threshold was determined similarly to the previous approach. We sampled the threshold from 0 to 1 and determined respective fidelity. Generalization from residue level towards classification on the structural level increased MCC values (Table 3).

| **Threshold** | **0** | **0.1** | **0.2** | **0.3** | **0.4** | **0.5** | **0.6** | **0.7** | **0.8** | **0.9** | **1.0** |
| --- | --- | --- | --- | --- | --- | --- | --- | --- | --- | --- | --- |
| **MCC** | 0.49 | 0.54 | 0.6 | 0.68 | 0.74 | 0.74 | 0.70 | 0.61 | 0.5 | 0.36 | 0.08 |

**Supplement Table 3**: Evaluation of the threshold parameter for classification of whole solenoid structures. The final threshold used in the leave one out analysis presented in the main paper was chosen to 0.5.

# Classification results for alternative classifiers

During the development of our method we experimented with different combinations of features (contacts and correlation coefficients) and classifiers (Decision Tree, SVM). The classifier presented in the main text is hence the best classifier we developed in the course of the research.

## Contact map features + SVM

We trained a SVM directly with feature vectors extracted from the CM for each residue. Hence, in opposite to the correlation features, these features were binary vectors. The extraction strategy was different to the one presented in the main text; values were extracted in the interval *[i;i+70]* for each residue *i*. Hence, all contacts above the maximum solenoid contact range (70 residues) were collected as a feature for each residue. However, the Mathews correlation coefficient was here determined to 0.33 at residue level.

|  | **Solenoid** | **Insert/NonSolenoid** |
| --- | --- | --- |
| Sensitivity | 35% | 91% |
| Precision | 67% | 74% |
| Accuracy | 73% | |
| MCC | 0.33 | |

**Supplement Table 4**: Evaluation of residue classification accuracies determined by SVM classification of contact patterns.

## Classification results of correlation + decision tree classifier

In another experiment the SVM classifier was replaced with a decision tree classifier based on the CART algorithm implemented in Scikit (Pedregosa & Varoquaux, 2011).

During the self-consistency experiment similar to the SVM training in the previous paragraph, we trained and tested the DT-classifier and were able to obtain almost perfect prediction for self-consistency tests. This result was, however, due to the fact that the default tree settings over-fitted to the dataset. Conclusively, we experimented with the tree parameters (tree height, leaf capacity) until self-consistency performed as bad as random prediction.

The further used training parameters for the CART tree were later chosen such that the maximum tree height should not exceed 100 nodes and that leafs should not contain less than 2 features. These settings still could be considered prone to over-fitting but already showed low accuracies in the self-consistency test. Leave-one-out classification led to the conclusion that using decision trees for this application was not as effective as the Correlation + SVM combination; the Mathews correlation coefficient of 0.4 and 0.59, respectively.

|  | **Solenoid** | **Insert/NonSolenoid** |
| --- | --- | --- |
| Sensitivity | 53% | 86% |
| Precision | 55% | 85% |
| Accuracy | 78% | |
| MCC | 0.40 | |

**Supplement Table 5**: Evaluation of residue classification accuracies determined by decision tree classification of correlation features.
