# Supplementary figures and images for: ConSole: using modularity of Contact maps to locate Solenoid domains in protein structures
Source: BMC Bioinformatics. 2014 Apr 27;15:119. doi: 10.1186/1471-2105-15-119 (PMC4021314; doi:10.1186/1471-2105-15-119)

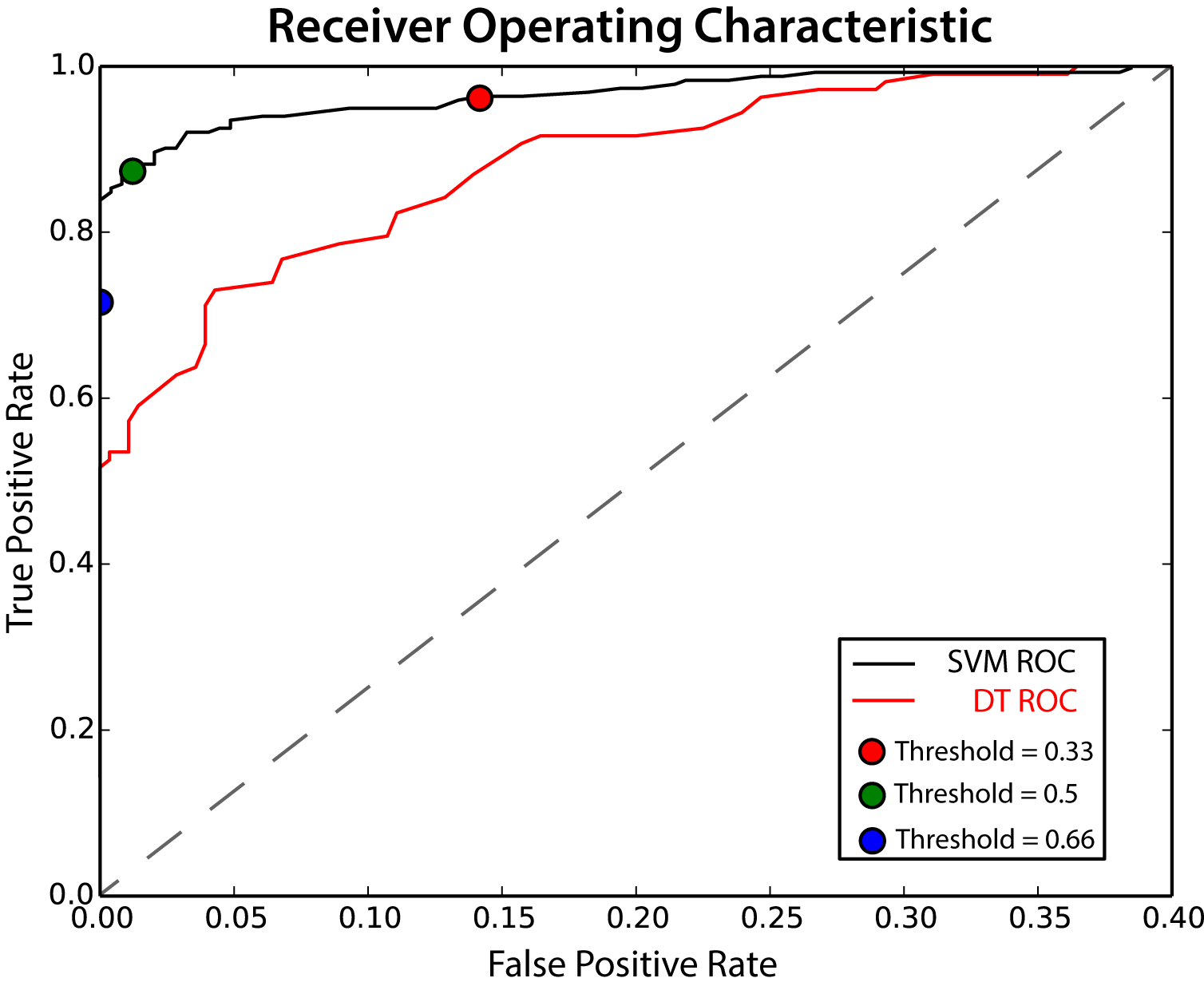

Supplement: Additional file 2: Figure S1 — ROC curve for our solenoid detection algorithms. A receiver operating characteristic curve determined for the classification of whole structures. For the support vector machine (main article) based classifier several thresholds scanned are marked on the black curve. The decision tree based classifier (red curve) performed significantly worse. [file 1471-2105-15-119-S2.png]
